# A Comparative Study of One-Stage Pre-pectoral Implant Breast Reconstruction With and Without Mesh

**DOI:** 10.7759/cureus.75896

**Published:** 2024-12-17

**Authors:** Hamed Hajiesmaeili, Shahram Shirazi, Kapil Agrawal, Raghavan Vidya

**Affiliations:** 1 Breast Surgery, The Royal Wolverhampton NHS Trust, Wolverhampton, GBR; 2 General Surgery, Croydon University Hospital, London, GBR; 3 Surgery, New Cross Hospital, Wolverhampton, GBR; 4 Surgery, The Royal Wolverhampton NHS Trust, Wolverhampton, GBR

**Keywords:** breast implant, implant-adm reconstruction, mastectomy, no adm, pre-pectoral reconstruction

## Abstract

Background

Pre-pectoral implant-based breast reconstruction has become increasingly popular because it is associated with less postoperative pain and earlier recovery than traditional sub-pectoral techniques. Acellular dermal matrix (ADM) in pre-pectoral reconstruction is thought to provide additional support for the implant and improve cosmetic outcomes. However, it leads to additional costs. This study aimed to compare the early outcomes of pre-pectoral implant-based breast reconstruction with and without mesh.

Methodology

An observational, single-surgeon, retrospective cohort analysis was conducted to evaluate patients who underwent one-stage pre-pectoral breast reconstruction between May 2019 and July 2023 at Royal Wolverhampton NHS Trust. Patient characteristics such as demographics, implant size, and postoperative complications were noted and compared. Statistical significance between groups was evaluated using chi-square tests, and a p-value <0.05 was deemed statistically significant.

Results

A total of 101 patients were included, with 52 patients with ADM and 49 patients without ADM. In total, 60 implant reconstructions were included in each group. Patients in the ADM group were younger than patients in the cohort without the mesh (median = 50 versus 56 years). Patients with ADM also had a higher median volume of breast implants than patients without mesh (430 vs. 330 cc). There were statistically more patients requiring postoperative radiotherapy in the ADM mesh group (p = 0.049). The early postoperative outcomes in both groups were comparable with no statistical differences in the rate of infection, seroma requiring aspiration, or implant loss.

Conclusions

This study which is one of the few studies comparing one-stage pre-pectoral implant reconstruction with and without mesh demonstrated that pre-pectoral reconstruction with no ADM is cost-effective and associated with comparable early postoperative outcomes. Our early observational series showed satisfactory outcomes; however, further studies are required to investigate longer-term and patient-related outcomes.

## Introduction

Breast cancer is the most common cancer among women in the United Kingdom, with at least 50,000 people in the United Kingdom being diagnosed every year. As mastectomy remains an important surgical treatment for many patients, post-mastectomy implant reconstruction is a vital part of maintaining psychological well-being and quality of life [1,2].

Pre-pectoral and sub-pectoral planes are the two main techniques for implant-based breast reconstruction [3]. Pre-pectoral breast reconstruction involves filling the space between the pectoralis major muscle and mastectomy skin flap. On the other hand, the sub-pectoral plane comprises lifting the pectoralis major muscle and placing the implant behind the chest wall [4].

The traditional reconstruction method of using the sub-pectoral plane was found to have a much longer recovery period and was associated with significant postoperative pain. Furthermore, the poor cosmetic outcomes associated with animation deformity were also a major hurdle associated with sub-pectoral implants [5,6]. Hence, the pre-pectoral plane has become the plane of choice for most surgeons for implant-based reconstruction considering the recent advances, especially in implant technology and better mastectomy techniques.

However, pre-pectoral reconstruction presents its own set of challenges, mainly concerning the support, stability within the skin envelope, and lower pole projection of the implant [7]. This is where the use of mesh, specifically acellular dermal matrix (ADM), comes into play. Mesh can provide additional support for the implant in a pre-pectoral plane and improve lower pole projection which can sometimes be challenging to achieve. However, studies have shown that despite these benefits the use of mesh may be associated with higher complication rates, including increased risk of infection and seroma formation [7-9]. Furthermore, it is important to note that mesh carries a significant cost which should be taken into consideration, especially in public healthcare settings.

Some surgeons believe that meshes have very little role in their practice and give importance to skin envelope design, implant choice, and, in some cases, the use of dermal slings to protect and stabilize the implant.

Given the ongoing debate and the mixed evidence regarding the necessity of mesh in pre-pectoral implant reconstruction, we conducted a single-center, single-surgeon, retrospective study comparing the outcomes of patients who underwent one-stage pre-pectoral implant reconstruction with and without ADM.

## Materials and methods

Study design

In this retrospective, comparative study of patients with pre-pectoral breast reconstruction with and without mesh, we obtained data from electronic records generated by our clinical web portal for patients at the Royal Wolverhampton NHS Trust between June 2019 and July 2023. A median follow-up period of 18 months was allocated. The study received local approval from the audit department in our institution.

Patient selection

Patients were included if they had undergone either nipple-sparing mastectomy or skin-sparing mastectomy followed by pre-pectoral breast reconstruction, which may have been performed with or without the use of ADM (specifically Braxon). Additionally, complete medical records needed to be available for review to qualify for study inclusion. Conversely, patients were excluded if they had insufficient follow-up data, underwent reconstruction using other techniques such as sub-pectoral reconstruction, or had a history of prior breast reconstruction surgery.

Technique

All patients underwent either a nipple- or skin-sparing mastectomy followed by pre-pectoral breast reconstruction. This technique, using Braxon mesh, has been described previously [10]. A similar technique was used for non-mesh-based implant breast reconstruction. For both techniques, we ensured that the mastectomy flaps were well vascularized, and diathermy use was minimized to preserve the vascularity of the flap. A minimum of one vacuum drain (14 F) was inserted, and prophylactic antibiotics were administered routinely for 14 days after surgery. Patients were discharged from the hospital with their drains in situ as per our unit’s standard practice. Drains were removed when the output was less than 20 mL per day over 48 hours.

Data collection

Data were collected from electronic medical records, encompassing a comprehensive range of demographic information, clinical details, and postoperative outcomes. Demographic variables included patient age, body mass index (BMI), and comorbidities. Clinical details recorded comprised the indication for mastectomy (cancer treatment or risk reduction), whether the reconstruction was unilateral or bilateral, and the use of adjunctive therapies such as radiotherapy. Early postoperative complications were also documented, including the rates and types of complications such as hematoma, infection, seroma requiring aspiration, superficial skin necrosis, wound dehiscence, and implant loss.

Statistical analysis

A quantitative statistical analysis of data was conducted using SPSS (IBM Corp., Armonk, NY, USA). Medians were calculated, and chi-square tests were used to determine any differences between the two groups of complications. A p-value <0.05 was deemed statistically significant.

## Results

In this study, we compared the demographic characteristics and complication rates of patients who underwent pre-pectoral breast reconstruction with and without mesh over an 18-month follow-up period. The data revealed several important findings, including a statistically significant difference in the rate of postoperative radiotherapy.

Demographic comparison

Each group included 60 implants. The breakdown of unilateral versus bilateral was similar, but the with-mesh group underwent a slightly greater number of unilateral procedures (44) compared to the without-mesh group (40). The median age of patients in the without-mesh group was higher at 56 years compared to the median age of 50 years in the with-mesh group. The median BMI was similar in the without-mesh group (26.72 kg/m^2^) compared to the with-mesh group (26 kg/m^2^).

Patients undergoing mastectomy for cancer were more common in the with-mesh group (46 patients) compared to the without-mesh group (37 patients). We noted that two patients in the with-mesh group underwent the procedure for risk reduction, while there were no risk reduction cases in the without-mesh group.

One of the most clinically significant findings was that the patients in the with-mesh group had a substantially increased rate of requiring postoperative radiotherapy (24 patients) compared to the without-mesh group (14 patients). This finding was statistically significant with a p-value of 0.049. The median size of the implant was also slightly larger with the use of mesh (430 cc) compared to the without-mesh group (330 cc) (Table 1).

**Table 1:** Demographic characteristics of with mesh and without mesh implant reconstruction.

	Without mesh (n = 49)	With mesh (n = 52)	P-value
Total implants	60	60	-
Unilateral	40	44	-
Bilateral	10	8	-
Median age	56	50	-
Median body mass index (kg/m^2^)	26.72	26	-
Mastectomy for cancer	37	46	0.089
Risk reduction	0	2	0.165
Postoperative radiotherapy	14	24	0.049
Median implant size (cc)	330	430	-

Complication rates

The analysis of complication rates revealed no statistically significant differences between the two groups for most complications. The occurrence of hematoma was slightly higher in the with-mesh group (two cases) compared to the without-mesh group (one case); however, this was not statistically significant (p = 0.558). Similarly, superficial skin necrosis was observed in two cases in the with-mesh group and one case in the without-mesh group, again without statistical significance (p = 0.558).

The red breast syndrome was reported in only one patient in the with-mesh group and none in the without-mesh group (p = 0.315). No difference was found between the rates of dehiscence, seroma, and implant loss in both groups (Table 2).

**Table 2:** Complication rates between the two groups.

	Without mesh	With mesh	P-value
Hematoma	1	2	0.558
Red breast syndrome	0	1	0.315
Superficial skin necrosis	1	2	0.558
Dehiscence	1	1	1
Seroma requiring aspiration	3	3	1
Implant loss	2	2	1
Hematoma	1	2	0.558

## Discussion

The evolution of breast reconstruction techniques has remarkably improved patient outcomes, especially with the availability of pre-pectoral implants. A recent study comparing submuscular and pre-pectoral implant placement after neoadjuvant chemotherapy established that pre-pectoral reconstruction is associated with reduced operative times, fewer muscle-related complications, and comparable complication rates to submuscular reconstruction, even in patients exposed to radiotherapy [11].

This study adds to the expanding conflicting literature on this technique by comparing pre-pectoral breast reconstruction with and without mesh and examining patient demographics, clinical outcomes, and complications. The resulting information is informative concerning the pros and cons of each approach and is likely to help with clinical decision-making.

The demographic analysis in this study revealed that younger patients were more likely to receive mesh-supported implants, with the median age in the with-mesh group being 50 years compared to 56 years in the without-mesh group. Furthermore, the larger median implant size in the with-mesh group (430 cc vs. 330 cc) suggests that mesh may be preferred when larger implants are used, potentially due to the added structural support mesh provides. The use of mesh may also assist with pocket control, preventing lateral migration of the implant and lower pole projection, especially in younger patients with larger breasts.

The notable finding that surgery for cancer with mastectomy was performed more frequently in the with-mesh group (46 patients) versus the without-mesh group (37 patients) suggests that mesh has a degree of bias toward therapeutic reconstructions as opposed to simple prophylactic or cosmetic reconstructions. This observation is not surprising as the literature supports that mesh may provide better aesthetics and implant stability in more complicated oncologic reconstructions [12].

However, it should be recognized that ADMs are not free from complications, with reports indicating that ADMs may be associated with a higher rate of infection, seroma, and red breast syndrome [13,14].

A notable finding in this study was the statistically significant higher rate of postoperative radiotherapy in the with-mesh group compared to the without-mesh group (p = 0.049). This observation suggests that mesh is more likely to be utilized when radiotherapy is anticipated in the treatment course. This association may be attributed to the presence of factors that simultaneously indicate the need for additional support, particularly in cases where radiotherapy might compromise the integrity of local tissues.

The literature has previously established that, in the context of implant reconstruction, radiation exposure is associated with an elevated risk of capsular contracture. One study reported that 40% of patients receiving radiation exposure subsequently require corrective surgery [15,16]. Furthermore, a meta-analysis by Awadeen et al. demonstrated that irradiated breasts were 2.89 times more likely to experience implant loss compared to non-irradiated breasts [17].

Although studies have suggested that ADMs can reduce capsular contracture through attenuating inflammatory signaling characteristics during the development of the breast capsule leading to thinner capsules [18], this no longer applies to patients undergoing radiotherapy, as reported by Spear et al. and Valdatta et al. [19,20]. Therefore, radiotherapy-related contracture in the ADM group will remain true and caution should be taken in monitoring these patients within the postoperative period.

Lipo-modeling or fat grafting is another technique that has gained universal acceptance to help rectify contour irregularities of the reconstructed breast. The method can be used to assist with capsular contracture, especially in patients undergoing radiotherapy, or to reduce the rippling effect of implants that are pre-pectoral, to improve cosmetic outcomes [21,22].

In this study, the comparison of complication rates between the two groups did not reveal any statistically significant differences for most of the complications examined, including hematoma, red breast syndrome, superficial skin necrosis, dehiscence, seroma requiring aspiration, and implant loss. These results are supported by current literature showing that pre-pectoral reconstruction without mesh is a safe and effective approach for implant reconstruction [12-23].

The cost-effectiveness of pre-pectoral implant-based breast reconstruction with and without ADM requires careful consideration of multiple factors. While our study did not directly measure costs, we can draw some inferences based on our findings and existing literature.

The most significant difference in direct costs between the two approaches is the price of the ADM itself. A preshaped 30 × 20 cm (600 cm^2^) Braxon mesh costs approximately £2,100. This substantial upfront expense is entirely avoided in non-mesh reconstructions, presenting a significant initial cost advantage.

Operative time is another critical factor influencing overall costs as operating theaters account for 9% of acute care costs in the NHS [9]. While our study did not measure this directly, other studies have reported on this aspect. Multiple studies indicate that the use of ADM typically adds 15-45 minutes to the operative time, with most estimates falling in the 20-30-minute range per breast [24,25]. Using the estimated operating theater cost of £14 per minute (based on a 2016 Wales Audit Office report), this additional time could translate to an additional cost of £210-£630 per breast for ADM-based reconstructions in addition to the existing material costs of the mesh itself [26].

One argument for ADM use is its potential to facilitate single-stage reconstruction. In select cases, ADM may allow for a larger permanent implant to be placed immediately, potentially avoiding the need for a two-stage expander/implant reconstruction [27]. A delayed reconstruction approach will incur material costs associated with a tissue expander, which is approximately £1,300 for saline versions. There are further costs associated with clinic appointments for periodic inflation and assessment of the prosthesis. Complications in these cases, such as infection, can further increase expenses through additional interventions, e.g., imaging, seroma aspiration, antibiotics, and hospital visits. Therefore, in carefully selected patients, the use of ADM mesh may be cost-effective in eliminating the need for a second surgery and reducing the overall treatment time despite the upfront material costs.

While our study was limited to an 18-month follow-up period, longer-term data from other studies can provide insights. As previously mentioned, some studies suggest that ADM use may lead to reduced rates of capsular contracture and fewer revisions over time, potentially offsetting the initial higher costs [28]. However, this needs to be balanced against reports of higher rates of certain complications with ADM use, such as infection and seroma, which could increase costs, which, while not demonstrated in this case series, have been reported by other surgeons [29].

Costs associated with postoperative complications, though not quantified in this cohort, may include additional outpatient follow-ups, the average cost of a single NHS outpatient appointment in breast surgery services according to the National Schedule of NHS Costs is £197, with an ultrasound-guided core needle biopsy of lesion of breast costing on average £49.69 [30].

It is our opinion that the decision whether to use mesh for pre-pectoral breast reconstruction should be specific to the individual patient, weighing factors such as age, size of the implant, skin envelope, oncological factors, and whether the patient is likely to require postoperative radiotherapy or chemotherapy. However, a careful design of the skin envelope with an appropriately sized or shaped implant may be just as effective in achieving satisfactory reconstruction without the added expense and financial implications of an ADM. Furthermore, other adjuncts such as lipo-modeling can be used to manage postoperative radiotherapy complications and deformity following pre-pectoral implant reconstruction.

It is important to note the limitations of this study. As this was a single-surgeon, retrospective study with a follow-up period of 18 months, it cannot capture long-term data regarding complications. Furthermore, the study did not address any aesthetic factors related to the implants, including rippling and patient-related satisfaction. Therefore, it is imperative to conduct prospective studies to examine the long-term outcomes of patients undergoing pre-pectoral implant reconstruction with and without ADM with a special emphasis on patient-related outcomes.

## Conclusions

This study which is one of the few studies comparing one-stage pre-pectoral implant reconstruction with and without mesh demonstrates that pre-pectoral reconstruction without ADM is cost-effective with comparable early postoperative outcomes. However, future research with a larger sample size and longer follow-up periods examining patient-reported outcomes to evaluate capsular contracture, aesthetic satisfaction, and viability of implants will further clarify the role of mesh/ADMs in pre-pectoral reconstruction, which can ultimately address this ongoing debate.
